# Editorial: Understanding the role of the autonomic nervous system in health and disease

**DOI:** 10.3389/fnins.2024.1446832

**Published:** 2024-06-26

**Authors:** Vitor E. Valenti, Luiz Carlos M. Vanderlei, Moacir F. Godoy

**Affiliations:** ^1^Autonomic Nervous System Center, School of Philosophy and Sciences, São Paulo State University, Marília, Brazil; ^2^School of Technology and Sciences, São Paulo State University (Unesp), Presidente Prudente, Brazil; ^3^Transdisciplinary Nucleus for the Study of Chaos and Complexity, NUTECC, São José do Rio Preto Medical School, FAMERP, São José do Rio Preto, Brazil

**Keywords:** autonomic nervous system, heart rate variability, sympathetic nervous activity, parasympathetic, vagus nerve

Human life depends on the autonomic nervous system (ANS). This is because the ANS controls involuntary body functions, such as blood pressure, gastrointestinal function, heart rate and bladder activity. The ANS is composed of a network which controls physiological systems through combined inputs from the internal and external environment to keep human body homeostasis. The central autonomic nervous network, the sympathetic and parasympathetic nervous systems regulates body functions (Wehrwein et al., 2016). Previous studies have reported the involvement of the ANS in human disorders (Affoo et al., 2012; Figueiredo et al., 2020; Kłysz et al., 2021) and also in good physical condition (Shivkumar and Ardell, 2016; Speer et al., 2024). Considering its importance for the human organism, this Research Topic grouped 86 researchers, 13 studies and provides recent data related to the role of the ANS in disease and health.

One paper conducted by Laurino et al., performed a crossover clinical trial to investigate the effects of hydration on heart rate variability (HRV) during and after aerobic exercise in patients with coronary artery disease. HRV is a non-invasive method that estimates autonomic function through fluctuations of heart interbeat intervals. Reduced HRV is usually related to abnormal adaptation of the ANS, indicating physiological malfunction, while increased HRV indicates good adaptation and is associated with healthy individuals with appropriate autonomic function (Sassi et al., 2015). The authors evaluated 38 men who performed two moderate-intensity aerobic exercise protocols, control and hydration protocols. As main findings, it was demonstrated that intake of eight portions of mineral water in a total amount proportional to the loss, administered during and after physical exercise was able to accelerate the recovery of non-linear HRV and that the fluid lost volume during exercise did not impact non-linear HRV.

Kirby et al. explored traditional time and frequency domain indices of HRV during autonomic dysreflexia after spinal cord injury. This experimental study was conducted in male Sprague Dawley rats submitted to spinal cord surgery to induce injury through a dorsal laminectomy at the T3 level. The results added integrated skin nerve activity as a biomarker that may be used for early detection and monitoring of autonomic dysreflexia.

HRV was also analyzed by Cheng W. et al. in 60 male inpatients with type 2 diabetes mellitus (T2DM) in sleep stages. The key questions asked whether HRV is more associated with metabolic function when sleep than during awake or the entire 24-h and if HRV changes during sleep cycles, inducing alterations in its interactivity with the metabolic system. Their data evidenced that metabolic function was associated with sleep quality, HRV differed between sleep and awake, indicating stronger and distinct interaction with metabolic function compared to 24-h and that HRV and its association with metabolic clinical indicators is altered during sleep cycles.

The heart rate rhythm autonomic control was investigated by Wu et al., who evaluated the acute effects of thoracic radiotherapy on HRV in 58 thoracic cancer patients. Participants were excluded in the presence of a pacemaker, those who previously received thoracic radiotherapy, patients with incomplete thoracic radiotherapy, and poor electrocardiogram quality. The main findings evidenced that thoracic radiotherapy significantly influenced the standard deviation of all normal RR intervals (SDNN), the root-mean square of differences between adjacent normal RR intervals (RMSSD), low frequency (LF), high frequency (HF) and total power HRV indices, suggesting reduced parasympathetic cardiac control and increased sympathetic cardiac regulation following this intervention.

The acute effects of an osteopathic technique were evaluated by Besson et al., who examined occipito-mastoid suture normalization intervention. The study examined 34 apparently healthy volunteers between 18 and 75 years old submitted to this passive and smooth osteopathic technique. The intervention led to higher RMSSD values compared to sham conditions, indicating increased parasympathetic cardiac control. However, the authors pointed to the short intervention period (10 min) applied in this study as a significant limitation, since conventional osteopathic technique interventions usually spend much more than 10 min.

The Poincaré plot was hypothesized by Yuan et al. as an index to predict the curative effect of metoprolol in pediatric postural orthostatic tachycardia syndrome. Based on their results, it was suggested that the longitudinal and transverse axis of the Poincaré plot can be used as an intuitive approach to anticipate the efficiency of metoprolol for postural orthostatic tachycardia syndrome. Nevertheless, the small sample size, the relatively basic graphic measurement methods and the single-center retrospective design were raised as relevant limitations.

In addition, Huang et al. examined traditional time and domain HRV indices in order to verify the effects of vagal nerve stimulation on esophageal motility and pharyngeal symptoms in patients with laryngopharyngeal reflux disease, which is a condition identified by stomach contents ejection into the larynx and pharynx, inducing inflammation in the throat (Mishra et al., 2020). As main results, it was found that acute transcutaneous auricular vagus nerve stimulation improved mental wellbeing and pharyngeal discomfort and increased upper esophageal sphincter pressures.

With this in mind, Wang et al. also investigated vagal nerve stimulation. The study evaluated the impact of transcutaneous auricular vagus nerve stimulation combined with task-oriented training on upper limbs activity in 20 subacute stroke patients compared to 20 participants in the sham group. The authors' data showed a positive effect of this intervention and suggested the modulation of cortical excitability as an explanation, since it may facilitate motor activity remodeling.

ANS function was investigated as a potential method for health complication prediction by Sharifi-Heris et al. The authors performed a prospective longitudinal observational study to analyze HRV during low risk pregnancy in healthy pregnant participants. The authors applied a hierarchical linear regression/mixed-effects model to understand the interaction between time as the independent variable and the root-mean square of differences between adjacent normal RR intervals (RMSSD) as the dependent variable to find out if the time could influence and estimate HRV. In conclusion, the study evidenced a distinction between healthy and complicated gestation according to the average HRV.

An interesting investigation of three cases published by Li et al. suggested a new treatment for restless leg syndrome. The study included a 47-years old male patient, a 57-years old male patient and a 24-years old female patient. Stellate ganglion block was proposed as a reliable method for treatment of symptoms, however, based on the small sample size, the authors recommended randomized controlled trials to provide further evidence regarding the effectiveness and safety.

The association between ANS and cognitive tasks was explored by Cheng S. et al. The study evaluated changes in ECG during distinct physical and cognitive loads during dynamic tasks in 35 healthy male volunteers. The n-back working memory task program was used as a cognitive load task and isotonic contraction of the left upper limb was used as a physical load procedure. Their main data pointed to the role of HRV during mental workload.

Another procedure explored in this Research Topic was acupuncture. Liu et al. tried to design a pain model by using HRV to comprehend the real-time changes in the ANS during acupuncture analgesia. This randomized, controlled, single-blind design evaluated 61 volunteers who received acupuncture intervention, 31 participants allocated to the sham group and 30 subjects in the model group. Interestingly, the authors found that acupuncture was related to increased heart rate vagal control. Moreover, the “needle sensation” presented positive correlation with both sympathetic and vagal heart rate regulation.

Finally, COVID was the focus of the study conducted by Barnden et al. The authors performed a pilot study to evaluate brain connectivity in post-COVID during cognitive tasks. The abovementioned cross-sectional investigation examined 10 long-COVID patients and 13 healthy control volunteers, which were submitted to functional MRI, cardiac and respiratory monitoring and the Stroop task. The data pointed to a number of differences in long COVID brain connectivity compared to healthy control participants. The differences related to the brainstem reticular activation system suggest that this structure may be crucial to long COVID sequel.

In summary, the articles published in this Research Topic present further elements related to HRV, hydration, sleep cycles, brain connectivity, metabolic function, cognitive function, long COVID, autonomic dysreflexia, spinal cord injury, restless leg syndrome, cognitive tasks and acupuncture. The data reported by this Research Topic provides valid evidence that adds relevant information to better understand the role of the ANS in disease and health.

## Author contributions

VV: Conceptualization, Data curation, Formal analysis, Investigation, Project administration, Resources, Supervision, Validation, Visualization, Writing – original draft, Writing – review & editing. LV: Conceptualization, Data curation, Formal analysis, Investigation, Visualization, Writing – original draft, Writing – review & editing. MG: Conceptualization, Data curation, Formal analysis, Investigation, Validation, Visualization, Writing – original draft, Writing – review & editing.
